# IL‐17A promotes cell migration and invasion of glioblastoma cells via activation of PI3K/AKT signalling pathway

**DOI:** 10.1111/jcmm.13938

**Published:** 2018-10-24

**Authors:** Qianqian Zheng, Shuo Diao, Qi Wang, Chen Zhu, Xun Sun, Bo Yin, Xinwen Zhang, Xin Meng, Biao Wang

**Affiliations:** ^1^ Department of Pathophysiology College of Basic Medical Science China Medical University Shenyang China; ^2^ Department of Neurosurgery First Affiliated Hospital of Dalian Medical University Dalian China; ^3^ Department of Geriatrics The First Affiliated Hospital of China Medical University Shenyang China; ^4^ Department of Neurosurgery The First Affiliated Hospital of China Medical University Shenyang China; ^5^ Department of Immunology College of Basic Medical Sciences of China Medical University Shenyang China; ^6^ Department of Urology ShengJing Hospital of China Medical University Shenyang China; ^7^ Center of Implant Dentistry School & Hospital of Stomatology China Medical University Shenyang China; ^8^ Department of Biochemistry and Molecular Biology College of Basic Medical Sciences of China Medical University Shenyang China

**Keywords:** glioblastoma, interleukin‐17A, invasion, migration, PI3K/AKT pathway

## Abstract

Glioblastomas (GBMs) are the most common of both benign and malignant primary brain tumours, in which the inflammatory and immunologic abnormalities are involved. Interleukin‐17A (IL‐17A) plays an important role in various inflammatory diseases and cancers. Several recent studies revealed that the expression of IL‐17A was overexpressed in human GBMs tissue. However, the accurate role of IL‐17A in GBMs remains unclear. In this study, we aimed to explore the effect of IL‐17A on cell migration and invasion of GBMs and the mechanism by which the effects occurred. We found that exogenous IL‐17A promoted significantly cell migration and invasion abilities in two GBMs cell lines (U87MG and U251) in a time‐dependent manner. In addition, the protein expressions of PI3K, Akt and MMP‐2/9 were increased in the GBMs cells challenged by IL‐17A. Furthermore, a tight junction protein ZO‐1 was down‐regulated but Twist and Bmi1 were up‐regulated. Treatment with a PI3K inhibitor (LY294002) significantly reduced the abilities of both migration and invasion in U87MG and U251 cells. LY294002 treatment also attenuated the IL‐17A causing increases of protein levels of PI3K, AKT, MMP‐2/9, Twist and the decreases of protein level of ZO‐1 in the U87MG and U251 cells. Taken together, we concluded that IL‐17A promotes the GBM cells migration and invasion via PI3K/AKT signalling pathway. IL‐17A and its related signalling pathways may be potential therapeutic targets for GBM.

## 1 INTRODUCTION

Glioblastoma, known as glioblastoma multiforme (GBM), is the most common type of primary malignant tumours in adult brain^1^, ^2^ with a mean survival rate ranging from 13.2 to 14.6 months^3^ and a 5‐year survival rate of 9.8%.^4^ Approximately 23 000 cases were newly diagnosed with GBM each year, and patients’ age has been identified as the most important prognostic factor for survival in patients with GBM in the United States.^5^, ^6^ Prognosis for patients with GBM is extremely poor because of high invasiveness of GBM. Thus, to elucidate the molecular mechanisms underlying the migration and invasion of GBM will be a prerequisite for providing novel therapeutic strategies against human GBM.

A large amount of studies tried to investigate the role of immune regulatory factors in GBM. Interleukin‐17A (IL‐17A) is produced by the main cytokine effector of T helper (Th17) cells and has been evoked the interest since the identification of Th17 in immunology.^7^, ^8^ Th17 cells, a recently discovered IL‐17A‐producing T cell subtype, have been reported in several extracranial and some intracranial tumours. IL‐17A has been shown to promote tumour development by several different mechanisms, including the enhancement of HIF‐1α expression induced by TNF‐α, the inhibited VASP expression^9^ and the activation of the IL‐6‐STAT3 signalling pathway.^10^ Recently, IL‐17A is reported to promote the migration and invasion of colorectal cancer cells by the up‐regulated the expression of MMP‐2/9 mediated by NF‐κB signalling.^11^ It improves the transition from chronic pancreatitis to pancreatic cancer through the novel REG3β‐JAK2‐STAT3 inflammatory pathway.^12^ IL‐17A is frequently observed in many cancers such as ovarian cancer,^13^ gastric cancer,^14^ and hepatocellular carcinoma,^15^ and being reported to associate with a poor clinical outcome in patients with GBM.^16^ Higher level of IL‐17A is found in glioma tissues as compared with trauma tissues,^17^, ^18^, ^19^ however, the role of IL‐17A in migration and invasion of GBM cells is not fully illuminated and the mechanism remains unclear.

In the present study, we found that IL‐17A expression was up‐regulated in human GBM tissues. Exogenous IL‐17A can increase cell motility of GBM cells and up‐regulate MMP‐2/9 expression via PI3K/AKT signalling pathway. These data indicate that IL‐17A signalling in gliomas may be the potential therapeutic target for GBM.

## 2 MATERIALS AND METHODS

### 2.1 GBM tissues and cell line

GBM tissues and adjacent non‐neoplastic brain tissues were obtained from GBM patients who underwent surgery at Shengjing Hospital of China Medical University. We obtained the patients’ prior written informed consent and ethics approval by the Institutional Research Ethics Committee of China Medical University for the use of clinical materials for research purposes. Glioma cell lines, U251 and U87, were maintained in the lab and the cells were cultured with Dulbecco's modified Eagle's medium (DMEM; Gibco, Carlsbad, CA, USA) containing 10% foetal bovine serum (FBS). Cells were incubated at 37°C in a humidified atmosphere with 5% CO_2_.

### 2.2 Immunohistochemistry

Briefly, the tissue sections were consecutively deparaffinized in xylene (I, II and III) and rehydrated using a graded series of alcohol (100% alcohol, 95% alcohol, 85% alcohol and 75% alcohol). Antigen retrieval process was performed in 0.01 M sodium citrate solution (pH 6.0) in a high‐pressure steam boiler for 10 minutes. Non‐specific binding was blocked by incubating the sections in phosphate‐buffered saline supplemented with 10% normal goat serum at room temperature for 1 hour. Immunoreactivity was evaluated separately by two experienced pathologists who were blinded to the clinicopathological data of the participants.

We used a staining index (SI; values 0‐12) with the following formula: SI = staining intensity × staining area, where intensities were scored semiquantitatively as follows: 0 (negative staining), 1 (mild staining), 2 (moderate staining) and 3 (intense staining). The staining area was scored as follows: 0, no staining of cells; 1, 1%‐25%; 2, 26%‐50%; 3, 51%‐75%; and 4, 76%‐100%. SI was graded as follows: 0‐1, negative expression; 2‐4, weakly positive expression; 5‐7, moderately positive expression and 8‐10, strongly positive expression. All of the three levels were considered as positive.

### 2.3 Reagents and antibodies

The human IL‐17A recombinant protein (rhIL‐17A, #8928), rabbit anti‐ZO‐1 polyclonal antibody (#8193), rabbit anti‐MMP‐9 polyclonal antibody (#15561), MMP‐2 antibody (#4022) the rabbit anti‐Slug polyclonal antibody (#9585), the rabbit anti‐AKT polyclonal antibody (#9272), the rabbit anti‐phospho‐AKT(Ser473) polyclonal antibody (#9271), the rabbit anti‐phospho‐PI3K polyclonal antibody (#4249), the rabbit anti‐phospho‐PI3K p85 (Tyr458)/p55 (Tyr199) polyclonal antibody (#4228), the anti‐rabbit IgG HRP‐linked antibody (#7074) and anti‐mouse IgG HRP‐linked antibody (#7076) were purchased from Cell Signaling Technology (Danvers, MA, USA). The mouse anti‐α‐SMA monoclonal antibody (ab7817), the mouse anti‐Twist monoclonal antibody (ab175430), the mouse anti‐Bmi1 monoclonal antibody (ab14389) were obtained from Abcam (Cambridge, UK). The mouse anti‐GAPDH monoclonal antibody was purchased from Beyotime Biotechnology (Shanghai, China). The Alexa Fluor^®^ 488 fluorescent second antibody and CellTracker CM‐Dil (C7000) were purchased from ThermoFisher Scientific (Waltham, MA, USA).

### 2.4 RNA isolation and quantitative real‐time PCR

Total RNA was extracted from GBM tissues with TRIzol™ reagent (TaKaRa, Kusatsu, Shiga, Japan) according to the manufacturer's instructions. The reaction was performed at 95°C for 5 minutes and then 40 cycles of 95°C for 15 seconds, 60°C for 1 minute, with a final extension at 95°C for 15 seconds, 60°C for 1 minute. Relative quantities (Δ cycle threshold (Ct) value) were obtained by subtracting the Ct value of IL‐17A from 18s. The fold change was calculated according to the formula 2^−ΔΔCt^. Each reaction was performed in triplicate. The sequences of forward and reverse primers are shown in Table 1.

**Table 1:** The sequences of forward and reverse primers

mRNA	Sequence
IL‐17A	
Forward	5‐CCACGAAATCCAGGATGCCCAAAT‐3
Reverse	5‐ATTCCAAGGTGAGGTGGATCGGTT‐3
18S	
Forward	5‐GCAGAATCCACGCCAGTACAAGAT‐3
Reverse	5‐TCTTCTTCAGTCGCTCCAGGTCTT‐3

### 2.5 Western blot

The cells were washed with ice‐cold PBS and lysed in a modified RIPA buffer containing 1 mM DTT, 1 mM PMSF, complete protease inhibitor cocktail (1 tablet/10 mL; Invitrogen, Carlsbad, CA, USA). The cell lysate was sonicated and followed by centrifugation at 12 000 *g* for 20 minutes at 4°C. Protein concentration was measured with BCA protein assay kit (Beyotime Biotechnology). Western blots were performed with specific antibodies to detect the corresponding proteins. After incubation at 4°C overnight, the blot was washed three times with 0.05% Tween‐20 TBS (TBST), and then incubated with 1:10 000 diluted goat anti‐rabbit/anti‐mouse IgG conjugated with HRP for 2 hours at room temperature. After additional washing with TBST, the target proteins on the blot membrane were visualized using the ECL system. The MF‐ChemiBIS 3.2 Imaging System (DNR Bio‐Imaging Systems, Jerusalem, Israel) was used for image capture. To control sampling error, the same blot was also probed for β‐Actin or GAPDH as an internal loading control. The integral optical density of each band was analysed using the Image‐J software and the ratio of band intensities of target protein over associated control was obtained as the statistic value. Data were expressed as the mean ± SD of at least three independent experiments.

### 2.6 MTT assay

U251 and U87 cells were seeded into 96‐well plates (5 × 10^3^ cells/well, 60% density) and challenged with rhIL‐17A at different concentrations. Then, 0.5 mg/mL MTT dye solution was added to each well and the cells were incubated at 37°C for 4 hours. Subsequently, the culture medium was discarded and 150 μL dimethyl sulphoxide was added to solubilize the precipitate. The absorbance was measured using a plate reader at 490 nm. Three dependent experiments were repeated. Data were presented as the mean ± SD.

### 2.7 Colony formation assay

The cells at a density of 1 × 10^3^ were seeded in 6‐well culture in culture medium with 10% FBS for 1 weeks. Then, the cells were fixed with methanol for 30 minutes and stained with 1% crystal violet for 10 minutes. Colonies of more than 50 cells were counted. All experiments were performed in triplicate. Data were presented as the mean ± SD.

### 2.8 Flow cytometry for the cell cycle assay

In brief, U251 and U87 cells were grown in 6‐well plates (5 × 10^5^ cells/well) challenged with rhIL‐17A with/without LY294002. Cells were harvested by exposure to trypsin/EDTA and centrifuged at 350 *g* for 5 minutes. Cell precipitates were washed three times with PBS. After fixation with 75% ethanol at 4°C overnight, each sample was washed again with PBS, and incubated with propidium iodide (100 mg/mL; Sigma, St. Louis, MO, USA) on ice for at least 30 minutes. Cell cycle fractions (G0/G1, S, and G2/M phases) were analysed by Flow Cytometry (FACS CantoTM II; BD BioSciences, San Jose, CA, USA). Proliferation index=(S+G2/M)/(G0/G1+S+G2/M). All experiments were performed in triplicate. Data were presented as the mean ± SD.

### 2.9 Wound healing assay

U251 and U87 cells were seeded in 24‐well culture plates (5 × 10^4^ cells/well). Twelve hours after treatment with rhIL‐17A, the cells were washed with PBS, and then scratches were made on the monolayer cells using a sterile P200 pipette tip to mimic the wound process. After removal of cell debris, the cells were observed under microscope to confirm the uniform width of scratches in each single group. The cells in the plate‐well were washed with PBS, and were incubated in DMEM containing 2% FBS. Five different zones of each well were chosen and the digital images were captured continuously (10× objective) from the same field at 0, 24 and 48 hours after scratching. This wound scratch assay was carried out in triplicate. All experiments were performed three times. Data were presented as the mean ± SD.

### 2.10 Transwell migration assay

For migration assay, the rhIL‐17A challenged U251 and U87 cells (4 × 10^4^ cells/well) were suspended in serum‐free culture medium and allowed to migrate for 12 hours. The chamber membranes with cells adhering to the lower surface were fixed with cold 4% paraformaldehyde for 20 minutes. All cells were stained with 0.2% crystal violet for 30 minutes, followed by washed three times with PBS and mounted on glass slides. Ten different fields of each membrane were selected randomly for the images capture. The percentage of migrated cells against total cells was calculated and used as cell migration index.

For invasion assay, the rhIL‐17A‐challenged U251 and U87 cells (8 × 10^4^ cells/well) were trypsinized and seeded in the upper chamber covered with Matrigel, whereas medium supplemented with 10% FBS was applied to the lower chamber. After incubation for 24 hours, the non‐invasive cells on the surface of the membranes were gently removed and the migrated cells on the lower side of the filters were fixed and stained with 0.2% crystal violet. Represent percentage of migrated cells was used as cell invasion index. All experiments were performed in triplicate. Data were presented as the mean ± SD.

### 2.11 Statistical analysis

All experiments were obtained at least in three replicates. In addition, assays producing quantitative data were run in triplicate. SPSS 19.0 software (IBM Corp, Armonk, NY, USA) was employed. Statistical significance was determined by one‐way ANOVA or two‐tailed Student's *t* test. The association of protein expression with clinicopathological features was analysed by the Pearson chi‐squared test. *P* < 0.05 was considered statistically significant.

## 3 RESULTS

### 3.1 Correlation between IL‐17A expression and clinical characteristics in GBM patients

**Figure 1:**
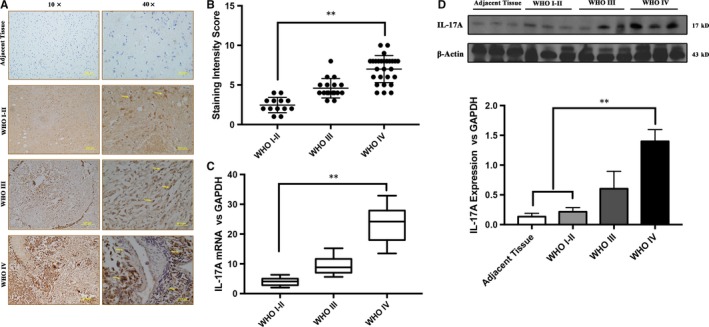
IL‐17A is overexpressed in glioma tissues. (A) IL‐17A protein expression was determined in adjacent tissue and glioma tissues (WHO I‐II, WHO III and WHO IV) by IHC. The data were shown at different magnification rates (20× and 40×). (B) IHC expression of IL‐17A quantified by staining score (0‐12) in GBM tissues and adjacent normal tissues. The levels of IL‐17A in GBM tissues are significantly higher than those in adjacent normal tissues. (C) Relative expression of IL‐17A mRNA in GBM tissues and paired adjacent tissues was determined by qPCR method. The mRNA level of IL‐17A in GBM tissues is significantly higher than that in adjacent normal tissues. (D) Expression levels of IL‐17A protein in three adjacent tissues, three WHO I‐II grade tissues and three WHO III‐IV grade tissues were determined with Western Blot method. Relative expression density of IL‐17A was quantified against β‐actin which was used as the inner control. IL‐17A expression was higher in GBM tissues than that in adjacent normal tissues. All data are shown as mean ± SD. *P < 0.05, **P < 0.01, compared with the adjacent normal group

Immunohistochemistry (IHC) staining assay was performed to examine the IL‐17A expression in paraffin‐embedded tissues from 57 GBM patients (WHO I+II: 13, WHO III: 17, WHO IV: 27) and six adjacent benign control tissues. The results showed that IL‐17A‐positive staining was significantly increased in WHO III‐IV glioma tissues compared to that in WHO I‐II glioma tissues and Adjacent tissues (**P < 0.01) (Figure 1A and B). In addition, the expression levels of IL‐17A in GBM tissues were evaluated by quantitative real‐time PCR (qPCR) and Western Blot assays. The results showed that mRNA expression of IL‐17A was significantly higher in WHO IV grade glioma tissues than that in WHO I‐II grade group. While protein expression of IL‐17A showed the same trend with the mRNA expression. (**P < 0.01) (Figure 1C and D).

The IL‐17A expression levels were positively correlated with the WHO pathological classification of GBM, but not with Age and Gender. In addition, the IL‐17A expression levels were markedly increased in WHO Grade III and Grade IV compared with that in WHO Grade I‐II (*P* = 0.011) (Table 2), indicating that IL‐17A might be a prognostic marker for GBM.

**Table 2:** Correlation between IL‐17A expression and clinicopathological characteristics

	Total (n = 57)	IL‐17A protein expression		*P*‐value
		Negative (n = 18)	Positive (n = 39)	
Age				
<55	34 (59.7%)	12 (35.3%)	22 (64.7%)	0.567
≥55	23 (40.3%)	6 (26.1%)	17 (73.9%)	
Gender				
Male	30 (50.9%)	10 (33.3%)	20 (66.7%)	0.784
Female	27 (49.1%)	8 (29.6%)	19 (70.4%)	
Tumour size				
<4.5 cm	31 (54.4%)	14 (45.2%)	17 (54.8%)	0.788
≥4.5 cm	26 (45.6%)	10 (38.5%)	16 (61.5%)	
Location				
Non‐eloquent	22 (38.6%)	9 (40.9%)	13 (59.1%)	0.787
Eloquent	35 (61.4%)	13 (37.1%)	22 (62.9%)	
Oedema				
None to mild	24 (42.1%)	9 (37.5%)	15 (62.5%)	0.584
Moderate to severe	33 (57.9%)	10 (30.3%)	23 (69.7%)	
Cystic change				
Absent	23 (40.3%)	11 (47.8%)	12 (52.2%)	0.414
Present	34 (59.7%)	12 (35.3%)	22 (64.7%)	
WHO grade				
I+II	13 (22.8%)	6 (46.2%)	7 (53.8%)	
III	17 (33.3%)	7 (41.2%)	10 (58.8%)	0.011
IV	27 (43.9%)	3 (11.1%)	24 (88.9%)	

Statistical analysis was carried out with Pearson chi‐squared test.

*P < 0.05 indicates a significant association between the variables.

### 3.2 IL‐17A promotes migration and invasion of GBM cells in vitro

**Figure 2:**
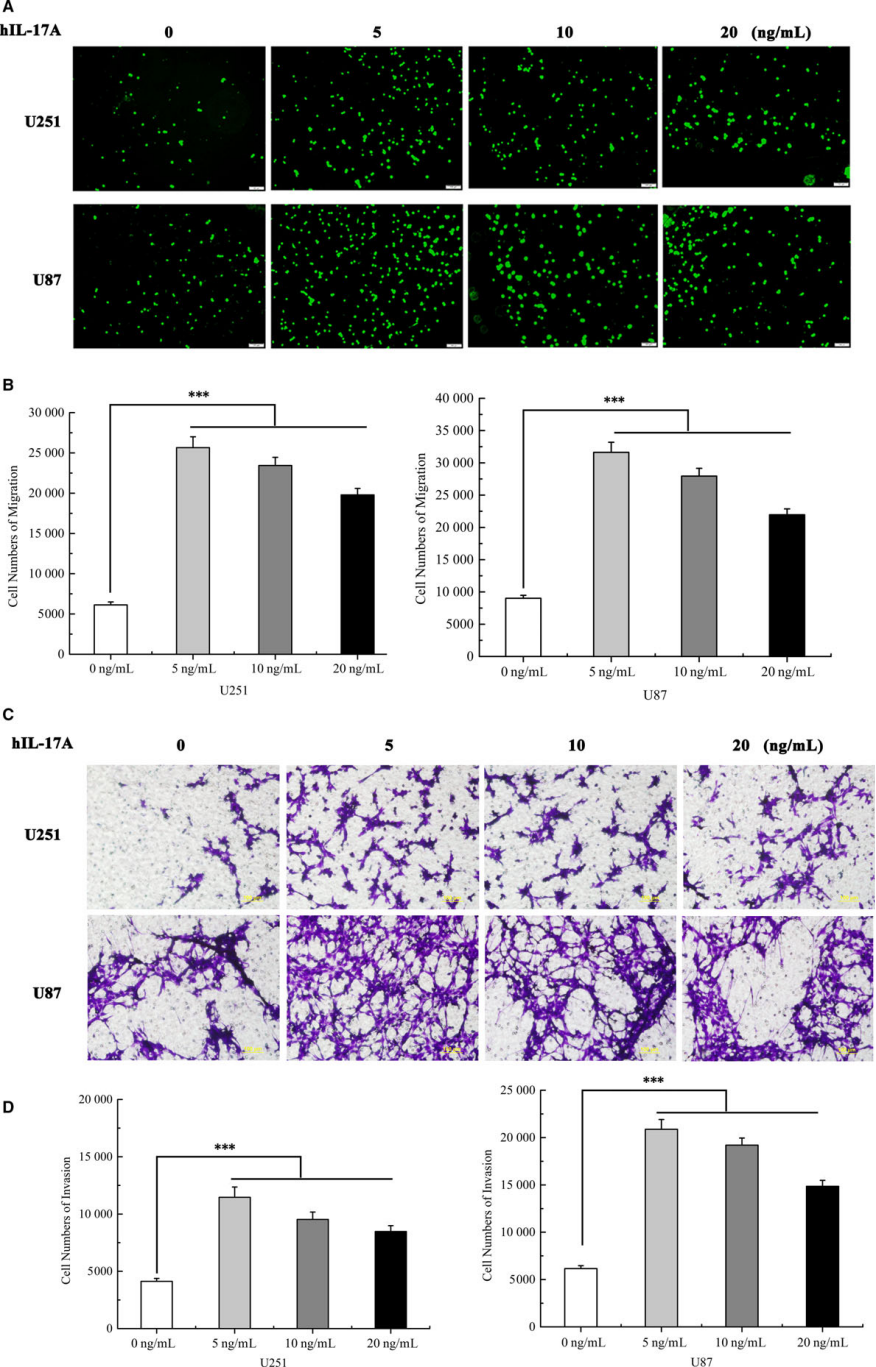
Effects of IL‐17A at different concentrations on cell migration and invasion of U251 and U87 cell lines. IL‐17A promoted cell invasion and migration in a dose‐dependent manner, which has been confirmed by (A) Transwell migration assay and (C) Matrigel Transwell invasion assay. Representative photomicrograph is presented. The number of migrated cells was counted and quantized (B and D). The difference between the control and the IL‐17 treated group was statistically significant. All data are shown as mean ± SD. *P < 0.05, **P < 0.01, compared with the control group

Previous studies showed that IL‐17A had an effect on cell migration and invasion in several type of cancers including lung cancer,^20^ colorectal cancer,^11^ gastric cancer^21^ and hepatocellular carcinoma.^22^ However, there is no report to show whether IL‐17A could promote GBM cell migration and invasion. Therefore, the effect of IL‐17A on cell motility was investigated by migration and by Matrigel invasion assays in U251 and U87 cells. The results showed that the mobility was significantly increased after IL‐17A treated at different doses (5, 10 and 20 ng/mL) compared with the control group (***P < 0.001) (Figure 2A and B). Moreover, the invasion assay showed that IL‐17A could also facilitate the invasion of the GBM cells (***P < 0.001) (Figure 2C and D). The results showed that 5 ng/mL concentration of IL‐17A achieved the most effective result. These data showed that IL‐17A could enhance GBM cells’ abilities of migration and invasion.

### 3.3 IL‐17A promotes activation of PI3K/AKT signalling pathway in glioma cells

**Figure 3:**
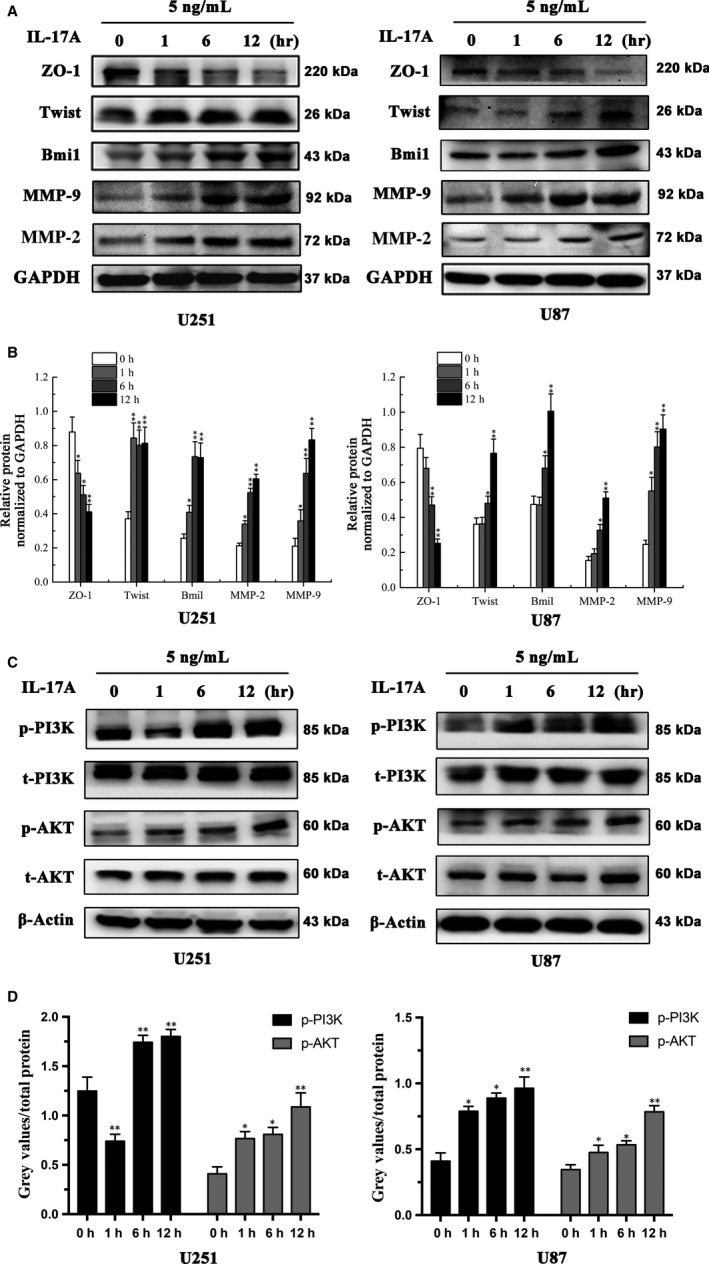
IL‐17 up‐regulated the expressions of migration biomarkers and activated PI3K/AKT pathway in a time‐dependent manner. (A) Western Blot analysis was carried out to detect the expression of ZO‐1, MMP‐2, MMP‐9, Twist and Bmi1 in U251 and U87 cells treated by IL‐17A (5 ng/mL) for 0, 1, 6, 12 h. β‐Actin was used as an inner control. (B) Quantification of the protein levels of ZO‐1, MMP‐2, MMP‐9, Twist and Bmi1 in glioma cells (*P < 0.05, **P < 0.01). (C) Western Blot analysis was carried out to detect the expression of t/p‐PI3K and t/p‐AKT in the glioma cells treated with IL‐17A (5 ng/mL) for 0, 1, 6, 12 h. β‐Actin was used as a loading control. (D) Quantification of the protein levels of t/p‐PI3K and t/p‐AKT in the glioma cells. All data are shown as mean ± SD. *P < 0.05, **P < 0.01, compared with the control group

MMP‐2/9 plays an important role in cancer metastasis,^23^ and IL‐17A is widely accepted as a regulator for MMP‐2 and MMP‐9.^22^ As expected, our result showed that IL‐17A up‐regulated the expressions of MMP‐9 and MMP‐2 in a time‐dependent pattern compared to the control group in U251 and U87 cells. ZO‐1 is a cell‐cell tight junction protein and a marker of cell motility^24^ While Twist and Bmi1 are also reported to closely correlate with migration and invasion of cancer cells.^25^, ^26^ IL‐17A treatment significantly decreased ZO‐1 expression but increased the expressions of Twist and Bmi1 in U251 and U87 cells compared to the untreated control group (*P < 0.05, **P < 0.01) (Figure 3A and B).

Akt activation promotes cellular growth and survival, and control of migration and invasion activities in GBM has been linked to activation of the PI3K/Akt pathway.^27^, ^28^ We hypothesized that IL‐17A may facilitate PI3K/Akt pathway activation in U251 and U87 cells. To confirm it, we treated glioma cells with IL‐17A (5 ng/mL) in a time course pattern. The result showed that phosphorylate levels of PI3K (Tyr458) and AKT (Ser473) were increased, while the total protein levels of PI3K and AKT were unchanged compared with control groups (*P < 0.05, **P < 0.01) (Figure 3C and D). Phosphorylation of AKT at Ser473 is necessary for its serine/threonine kinase activity, and also correlates with metastatic properties of several cancer cell lines. As shown in the result, IL‐17A up‐regulated the metastasis markers, such as Twist, Bmi1 and MMPs and down‐regulated the junction marker ZO‐1 expression. In addition, IL‐17A could activate the PI3K/AKT pathway. This gave us a hint that IL‐17A induced GBM cells migration and invasion could be through the PI3K/AKT pathway.

### 3.4 IL‐17A prompts glioma cells motility in a PI3K/AKT‐dependent pathway

**Figure 4:**
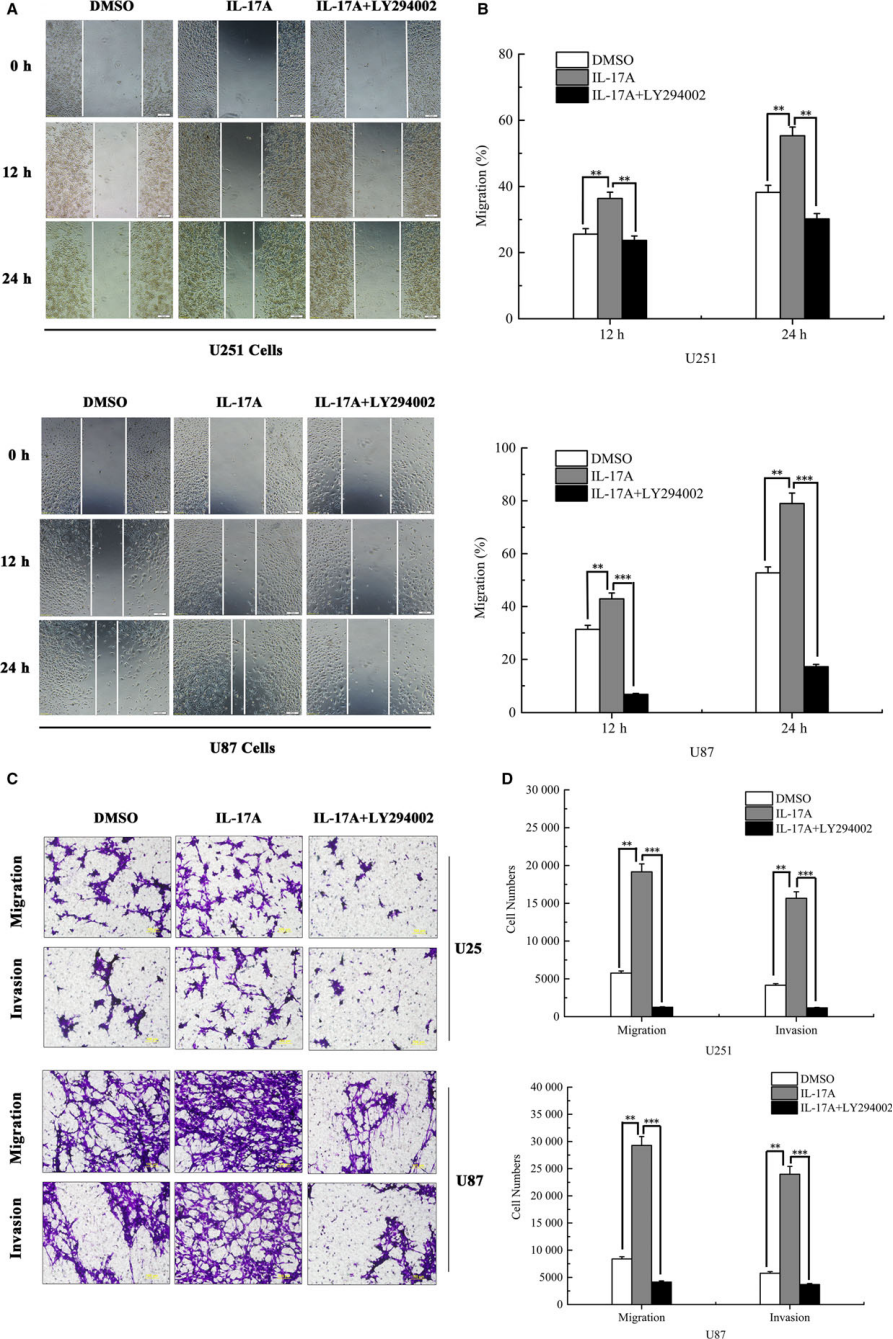
IL‐17A promoted cell migration and invasion of U251 and U87 cell lines. (A) Wound healing assay and (C) Matrigel Transwell invasion assay showed that IL‐17A (5 ng/mL) significantly promoted the migration and invasion of U251 and U87 cells compared with the control group. PI3K inhibitor (LY294002) abolished the migration and invasion capability induced by IL‐17A. Representative photomicrograph is presented. (B and D) The number of migration cells were calculated and quantitative. The differences between IL‐17‐induced group and with/without LY294002 were statistically significant. All data are shown as mean ± SD. *P < 0.05, **P < 0.01, compared with the control group

To address the effect of PI3K/AKT signalling pathway on IL‐17A‐induced MMP‐2/9 expression and cell invasion, U251 and U87 cells were pretreated with LY294002 (20 μM), a PI3K inhibitor, for 12 hours and then continue to incubate with rhIL‐17A (5 ng/mL) for 12 hours. Wound healing assay and Transwell migration/invasion assays showed that IL‐17A‐induced cell motility and invasiveness were markedly attenuated by LY294002 in vitro. In order to further discuss whether the PI3K/AKT pathway affects ZO‐1, Twist and MMP‐2/9 production in GBM cells treated with IL‐17A, a PI3K/AKT inhibitor (LY294002) was used to treat U251 and U87 cells before IL‐17A was applied. The inhibition is specific for PI3K at concentrations up to 20 μM. The results showed that treatment with LY294002 significantly abolished the effect of IL‐17A on migration (**P < 0.01, ***P < 0.001) (Figure 4A and B) and invasion (**P < 0.01, ***P < 0.001) (Figure 4C and D) of U251 and U87 GBM cells.

### 3.5 IL‐17A up‐regulated Twist and MMP‐2/9 expressions through PI3K/AKT pathway activation

**Figure 5:**
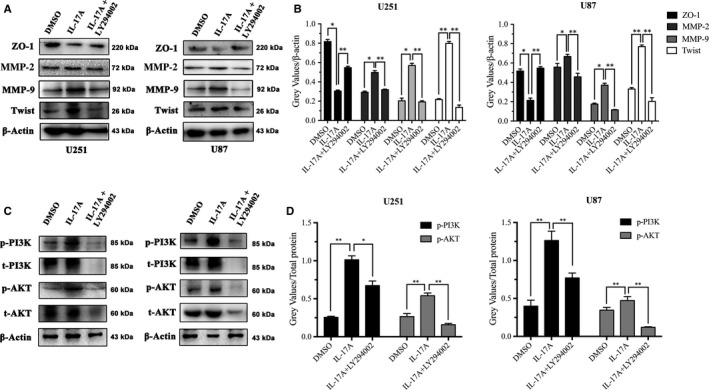
IL‐17 up‐regulated migration markers expression via PI3K/AKT pathway. (A) Western Blot analysis was used to detect the expressions of ZO‐1, MMP‐2/9 and Twist in U251 and U87 cells treated by IL‐17 (5 ng/mL) with/without LY294002. β‐Actin was used as a loading control. (B) Quantification of the protein levels of ZO‐1, MMP‐2/9 and Twist in glioma cells. (C) Western Blot analysis was carried out to detect the expressions of t/p‐PI3K and t/p‐AKT in the glioma cells treated with IL‐17A (5 ng/mL) with/without LY294002. β‐Actin was used as a loading control. (D) Quantification of the protein levels of t/p‐PI3K and t/p‐AKT in the glioma cells. All data are shown as mean ± SD. *P < 0.05, **P < 0.01, compared with control group

U251 and U87 cells were treated with LY294002 (20 μM) before IL‐17A treatment, and the expressions of ZO‐1, MMP‐2/9 and Twist were reversed compared to IL‐17A treated only (*P < 0.05, **P < 0.01) (Figure 5A and B). In addition, Western Blot analysis revealed that pretreatment with LY294002 abolished the IL‐17A induced up‐regulation of PI3K and AKT either total protein levels or phosphorylated protein levels respectively (*P < 0.05, **P < 0.01) (Figure 5C and D). These results were similar to the results from previous studies using the same inhibitor.^29^, ^30^, ^31^, ^32^, ^33^ Taken together, the results demonstrated that the IL‐17A promoted GBM cell migration and invasion were dependent on MMP‐2/9 and Twist via activating PI3K/AKT signalling pathway.

### 3.6 IL‐17A promoted glioma cells proliferation via PI3K/AKT pathway

**Figure 6:**
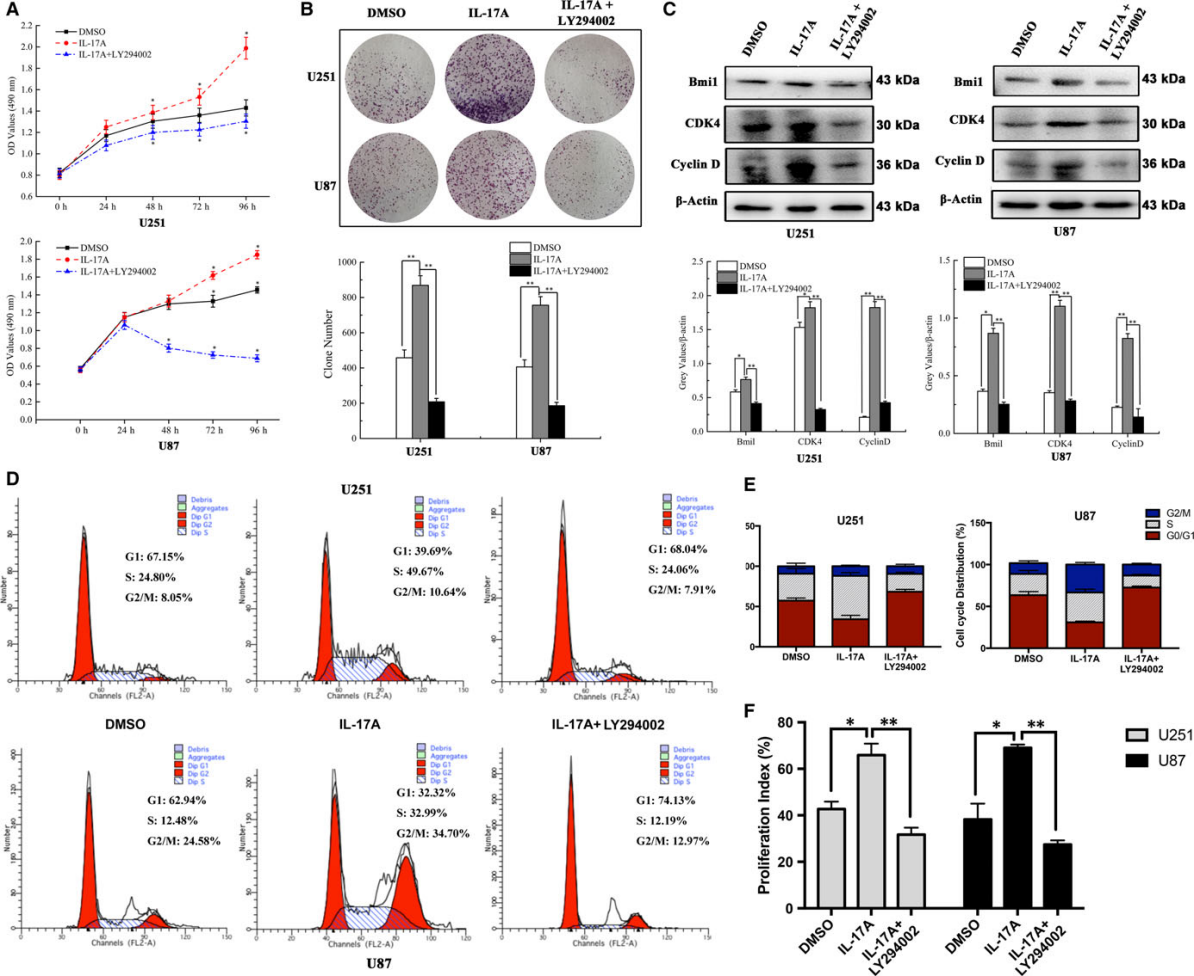
Role of PI3K/AKT pathway in the cell growth of U251 and U87 cells treated with IL‐17A. (A) MTT assay showed the cell viabilities of U251 and U87 cells treated with IL‐17A (5 ng/mL) with/without LY294002 for different periods (24, 48, 72 and 96 h). (B) A colony formation assay was used to determine the self‐renewal effect of IL‐17A‐induced in U251 and U87 cells. The colonies were counted. The number of the colonies was calculated and quantized. The difference between IL‐17‐treated group and the control group or (Inhibitor of PI3K) LY294002 group were statistically significant. (C) Western blot results confirmed that Bmi1, CDK 4 and Cyclin D1 were up‐regulated in the IL‐17‐treated group compared with control group. In addition, LY294002 could reverse this result. Quantification of the protein levels of Bmi1, CDK 4 and Cyclin D1 in glioma cells. (D) Representative cell cycle images and (E) histograms of U87 and U251 cells treated with IL‐17A with or without LY294002 were given and (F) proliferation index (PI=(S+G2/M)/(G0/G1+S+G2/M) were analysed by flow cytometry analysis. All data are shown as mean ± SD. *P < 0.05, **P < 0.01, compared with control group

Since PI3K/AKT pathway could modulate cell proliferation and a study reported that IL‐17A overexpression promoted U87 tumourigenesis in nude mice.^34^ For this reason, we further examined the effect of IL‐17A on GBM cells growth in vitro. Using a MTT assay, we found that cell growth was much faster in U251 and U87 treated with IL‐17A than that of the control group. Interestingly, LY294002 inhibited the cell proliferation effect of IL‐17A (*P < 0.05) (Figure 6A). The result of colony formation assay further showed that IL‐17A promoted U251 and U87 cells proliferation, while pretreatment with LY294002 reversed the cell proliferation effect of IL‐17A (**P < 0.01) (Figure 6B). Because Bmi1 is a key modulator in invasion, proliferation and self‐renewal in GBM^35^ Western Blot analysis was carried out subsequently to detect the expressions of Bmi1 as well as CDK4 and Cyclin D in the IL‐17A treated cells with/without LY294002. The results showed that Bmi1, CDK4 and Cyclin D were up‐regulated by IL‐17A, while the effect was inhibited when treated with PI3K/AKT pathway inhibitor LY294002 (Figure 6C). Flow cytometry was used to analyse the cell cycle phase distribution, and the results showed the increased cell number in S phase in the cells challenged by rhIL‐17A compared with that of control group and the effect was reversed by LY294002 (Figure 6D and E). The proliferation index reflects the ratio of S phase and G2/M phase of the cell cycle in the U251 and U87 cells (Figure 6F). Taken together, the data suggested that IL‐17A could promote cell growth of GBM cells via PI3K/AKT pathway (*P < 0.05, **P < 0.01).

## 4 DISCUSSION

Tumour microenvironment is composed of multiple surrounding cells (eg, immune cells and fibroblasts), signalling molecules and the extracellular matrix.^36^, ^37^ The tumour cells can communicate with the surrounding microenvironment while the immune cells can interact with tumour cells by direct contact or secreting different cytokines,^38^ functioning as a double‐edged sword for cancer cell growth or death. Among these cytokines, IL‐17A has been attracted much more attention. As a pro‐inflammatory cytokine secreted primarily by Th17 cells, IL‐17A has been found frequently to be involved in many cancer entities, such as ovarian cancer,^39^ pancreatic cancer,^40^ tongue squamous cell carcinoma,^41^ cervical cancer^42^ and hepatocellular carcinoma.^22^

Malignant GBMs are primary tumours of the central nervous system characterized by diffuse infiltration into the brain, which are the one of most challenging malignancies to treat in clinic.^43^ Although there are several studies carried out to investigate the potential role of IL‐17A in the progression of malignant GBMs, the mechanism of IL‐17A could influence glioma progression remains unclear. In GBM patients, high IL‐17A infiltration is associated with poorer prognosis, suggesting it as a potential prognosis factor.^16^ Previously, it has been reported by other group that IL‐17A protein expression is increased in traumatic brain injury group compared with the sham‐operated group in a rat model.^44^ As a pro‐inflammatory factor, the IL‐17A expression is weak in normal tissues but much strong in the trauma tissue. Wainwright et al found mRNA expression of IL‐17A in human GBM^18^ and Hu et al further confirmed the higher expression of IL‐17 mRNA in 18 tumour tissues derived from out of 24 patients with malignant glioma than that in tissues derived from trauma patients, but there was no difference among various histologic grades of GBM.^17^ On the contrary, our results showed that IL‐17A expressions were significantly higher in WHO IV grade GBMs tissues than those in the WHO III, WHO I‐II and adjacent tissues. Meanwhile, the expression of IL‐17A in tumour specimen from patients with GBM had the parallel result to the protein and mRNA levels of IL‐17A. The increases of IL‐17A in advanced grade indicated the potential correlation between the IL‐17A expression and GBM metastasis because GBM cells exhibited an invasive growth pattern even in the low‐grade (WHO I‐II) of well‐differentiated cells. It was proved that IL‐17A could promote migration and invasion of several kinds of cancer, but there was no published report on role of IL‐17A in migration and invasion of GBM cells. Here, we investigated the migration and invasion abilities in human GBM cell lines and found that IL‐17A promoted migration and invasiveness of U251 and U87 glioma cell lines, similar to previous reports. However, higher concentration of hIL‐17A did not show significantly the enhanced ability of cell migration and invasion compared with lower concentration. Interestingly, we found a decrease trend from 5 to 20 ng/mL treatment. A previous study shows a similar result of ours, a low concentration of IL‐17A could enhance osteoclast precursors’ autophagy, whereas a high level of IL‐17A is contrary.^45^ Nevertheless, the result showed that cells acquired an ability of migration and invasion when treated with hIL‐17A from 5 to 20 ng/mL as shown in the Transwell assays.

MMP is a key factor for tumour invasion and metastasis, which catalyse the degradation of the extra cellular matrix.^46^ Thus, we further investigated whether MMPs were involved in the GBM cell migration and invasion induced by IL‐17A. Our results revealed that IL‐17A treatment significantly increased the expression of both MMP‐2 and MMP‐9 at protein levels, indicating that up‐regulated expression of MMP‐2 and MMP‐9 could be responsible for this invasive behaviour of cell treated with IL‐17A. Similarly, previous studies also proved that IL‐17A could increase cellular motility in many types of cancer, including lung cancer,^47^ hepatocellular carcinoma^22^ and gastric cancer,^48^ via activating MMP‐2/9. However, the mechanisms underlying IL‐17A‐induced up‐regulation of MMPs expression in GBMs is different from other cancers.

As the well‐known vital transcription factors, Twist and Bmi1 play an important role in metastasis and their dysregulation has been demonstrated in metastatic cancers. We found that IL‐17A increased the expressions of both Twist and Bmi1 at protein level, which could be one of the major reasons for the enhanced invasion and migration in U87 and U251 induced by IL‐17A treatment. Twist and Bmi1 are also both involved in the proliferation and tumourigenesis of cancers. It was reported that Twist1 was involved in progression of human gastric cancer, promoting the proliferation of gastric cancer cells.^49^ Bmi1 knockdown resulted in inhibition of clonogenic potential in vitro and brain tumour formation in vivo,^30^ and inhibition of proliferation and invasion in human bladder cancer cells.^26^ In this study, we examined the role of IL‐17A in proliferation of GBM cells and the expressions of cell cycle‐related proteins, in vitro. We found that IL‐17 promoted cell growth of GBM cells and obviously increased the protein expressions of CDK4 and Cyclin D. Then, we explored the underlying signalling pathway regarding IL‐17A function as a pro‐metastatic and pro‐proliferative factor. PI3K/Akt pathway was a well‐known classic signal transduction pathway involved in cell growth, proliferation, motility, survival and apoptosis. Abnormal activation of the PI3K/Akt pathway resulted in the survival and proliferation of tumour cells in various human cancers.^50^ Our results showed that IL‐17A increased both phosphorylated protein levels of PI3K (Tyr458) and AKT (Ser473), indicating the activation of PI3K/AKT pathway. Meanwhile, our results also showed that a PI3K inhibitor LY294002 substantially reduced not only increased abilities of invasion and migration induced by IL‐17A, but also the protein expressions of MMP‐2/9, Twist and Bmi1 in U87 and U251 cells. Furthermore, PI3K inhibitor LY294002 hindered the enhanced proliferation of U87 and U251 cells treated by IL‐17A, restoring the protein expressions of CDK4 and Cyclin D.

In this study, we aimed to explore the effect of hIL‐17A on GBM cells. However, the in vivo function of IL‐17A needs to be proved and verified using animal models of GBM. In addition, it is most important to clarify and determine the potential functions of IL‐17A in the tumour tissues derived from GBM patients.

**Figure 7:**
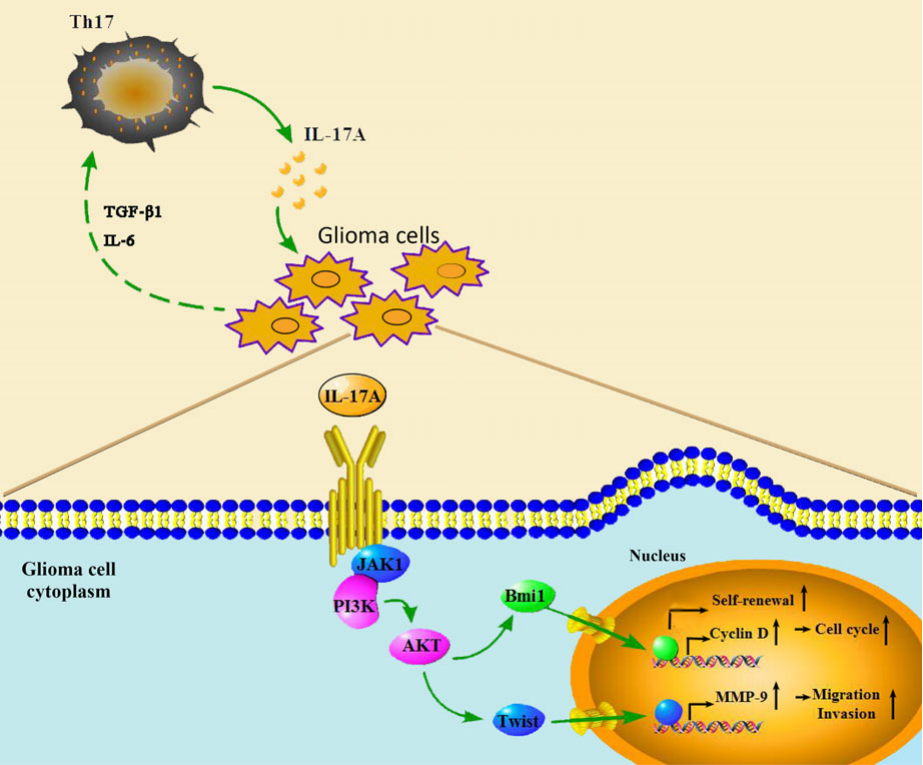
Working model. IL‐17A functions as a pro‐metastatic and pro‐proliferative factor in glioma cells via activation of PI3K/AKT signalling pathway

Here, we demonstrated that IL‐17A functioned as a pro‐ metastatic and pro‐ proliferative factor, in vitro. We revealed the molecular mechanisms that IL‐17A induced GBM cell invasiveness through the PI3K/AKT mediated MMP‐2/9 activation (Figure 7). In conclusion, our study provided evidence that IL‐17A could promote the migration and invasion of GBM cells. Considering that tumour metastasis is often associated with poor prognosis and high mortality among GBM patients, our study is clinically relevant and IL‐17A signalling could be a novel target for GBM therapy, especially GBM metastasis. Furthermore, serum IL‐17A level could be potentially a valuable marker in GBM patients who were at high risk of metastasis.

## CONFLICT OF INTEREST

The authors declare that they have no competing interests.
